# Exploring the therapeutic potential of an antinociceptive and anti-inflammatory peptide from wasp venom

**DOI:** 10.1038/s41598-023-38828-w

**Published:** 2023-08-01

**Authors:** Priscilla Galante, Gabriel A. A. Campos, Jacqueline C. G. Moser, Danubia B. Martins, Marcia P. dos Santos Cabrera, Marisa Rangel, Luiza C. Coelho, Karina S. Simon, Veronica M. Amado, Jessica de A. I. Muller, Johannes Koehbach, Rink-Jan Lohman, Peter J. Cabot, Irina Vetter, David J. Craik, Monica C. Toffoli-Kadri, Victoria Monge-Fuentes, Jair T. Goulart, Elisabeth F. Schwartz, Luciano P. Silva, Anamelia L. Bocca, Márcia R. Mortari

**Affiliations:** 1 Laboratory of Neuropharmacology, Department of Physiological Sciences, University of Brasília, Brasília, DF 70910-900 Brazil; 2 Department of Physics, IBILCE, São Paulo State University, São José do Rio Preto, SP 15054-000 Brazil; 3 Immunopathology Laboratory, Butantan Institute, Sao Paulo, SP 05503-900 Brazil; 4 Laboratory of Nanobiotechnology, Embrapa Genetic Resources and Biotechnology, Brasília, DF 70770917 Brazil; 5 Laboratory of Applied Immunology, Department of Cell Biology, University of Brasilia, Brasilia, DF 70910-900 Brazil; 6 Faculty of Medicine and University Hospital of Brasília, University of Brasilia, Brasilia, DF 79910-900 Brazil; 7 Laboratory of Pharmacology and Inflammation FACFAN, Federal University of Mato Grosso do Sul, Campo Grande, Mato Grosso do Sul 79070-900 Brazil; 8 Institute for Molecular Bioscience, Australian Research Council Centre of Excellence for Innovations in Peptide and Protein Science, The University of Queensland, Brisbane, QLD 4072 Australia; 9 School of Pharmacy, The University of Queensland, Brisbane, QLD 4072 Australia

**Keywords:** Drug discovery, Neuroscience, Biotechnology

## Abstract

Animal venoms are rich sources of neuroactive compounds, including anti-inflammatory, antiepileptic, and antinociceptive molecules. Our study identified a protonectin peptide from the wasp *Parachartergus fraternus*' venom using mass spectrometry and cDNA library construction. Using this peptide as a template, we designed a new peptide, protonectin-F, which exhibited higher antinociceptive activity and less motor impairment compared to protonectin. In drug interaction experiments with naloxone and AM251, Protonectin-F's activity was decreased by opioid and cannabinoid antagonism, two critical antinociception pathways. Further experiments revealed that this effect is most likely not induced by direct action on receptors but by activation of the descending pain control pathway. We noted that protonectin-F induced less tolerance in mice after repeated administration than morphine. Protonectin-F was also able to decrease TNF-α production in vitro and modulate the inflammatory response, which can further contribute to its antinociceptive activity. These findings suggest that protonectin-F may be a potential molecule for developing drugs to treat pain disorders with fewer adverse effects. Our results reinforce the biotechnological importance of animal venom for developing new molecules of clinical interest.

## Introduction

Throughout millions of years of evolution, some animals have developed the capacity to produce and store a series of biologically active compounds known as venoms^1,2^. The use of venom is found in approximately 100,000 species of animals distributed in the most diverse phyla. Venomous animals have emerged independently about 100 times throughout the evolutionary process^3^, evidencing the various ecological advantages conferred by the use of venom for predation and defense.

Despite causing extensive systemic effects, Hymenoptera venom has been used in therapy for thousands of years. Reports describe the use for pain and inflammation control in Ancient Egypt and Traditional Chinese Medicine (for review, see^4^). In a double-blind randomized controlled trial, authors have described clinical evidence of the efficacy and safety of acupuncture use associated with bee venom in patients with chronic pain^5^. These data reveal the immense potential that the study of neuroactive compounds isolated from Hymenoptera brings for the treatment of pain.

Among the Hymenoptera, wasp venom is relatively less studied, especially the neuroactive compounds it contains. The major bioactive compounds found in the venom of wasps are peptides, with molecular mass ranging from 1 to 7 KDa, which can comprise about 70% of the dry weight of the crude venom^6^. With a very different defense behavior, the eusocial and tropical wasp *Parachartergus fraternus* has the ability to paralyze prey, an uncommon behavior for social wasps and more frequent for solitary wasps^7^. The ability to paralyze prey, exhibited by some invertebrate animals, results from the action of potent neuroactive compounds with activities at various molecular targets such as voltage-dependent or ligand-dependent ion channels^8^. Therefore, the study of the composition and effect of venom components may reveal molecules of pharmacological relevance with neurobiological activity, especially for the control and relief of pain^9^.

Pain is a complex multidimensional event associated with a protective response of the organism in the presence of harmful stimuli from the external environment or from the organism itself and may be directly or indirectly associated with a neurological disorder^10^. Chronic pain represents is a public health problem that affects one-third of the world's population^11^. Currently, the drug most used in the clinic is the sulfate morphine, however, the opioid use disorders are vastly unpleasant (addiction, constipation, tolerance, overdose, and occasionally death), affecting over 16 million people worldwide. In special, the more serious and widespread adverse effect, the tolerance affects the safety and comfort of patients, and it causes clinically significant distress or impairment^12^.

Thus, the objective of the research was to isolate and identify an antinociceptive peptide from the venom of the social wasp *P. fraternus* and evaluate its modulation of inflammatory response. From the sequence of this natural peptide, identified as a protonectin, we used rational peptide design as a biotechnological tool to develop a novel peptide with a more potent antinociceptive effect, named protonectin-F. Its biological effects and mechanism of action were further characterized through several biological assays, showing promising applications for pain relief at the clinical level.

## Results

### Collection and extraction of the venom

Two nests of *Parachartergus fraternus* were collected to obtain the glands and reservoirs, rendering a total of approximately 2,500 individuals and 2,380 glands. After extraction, filtration, and drying, 240 mg of low molecular weight compounds (LMWC up to 3,000 Da) were obtained.

### Fractionation and mass spectrometry of the peptide of P. fraternus

**Figure 1:**
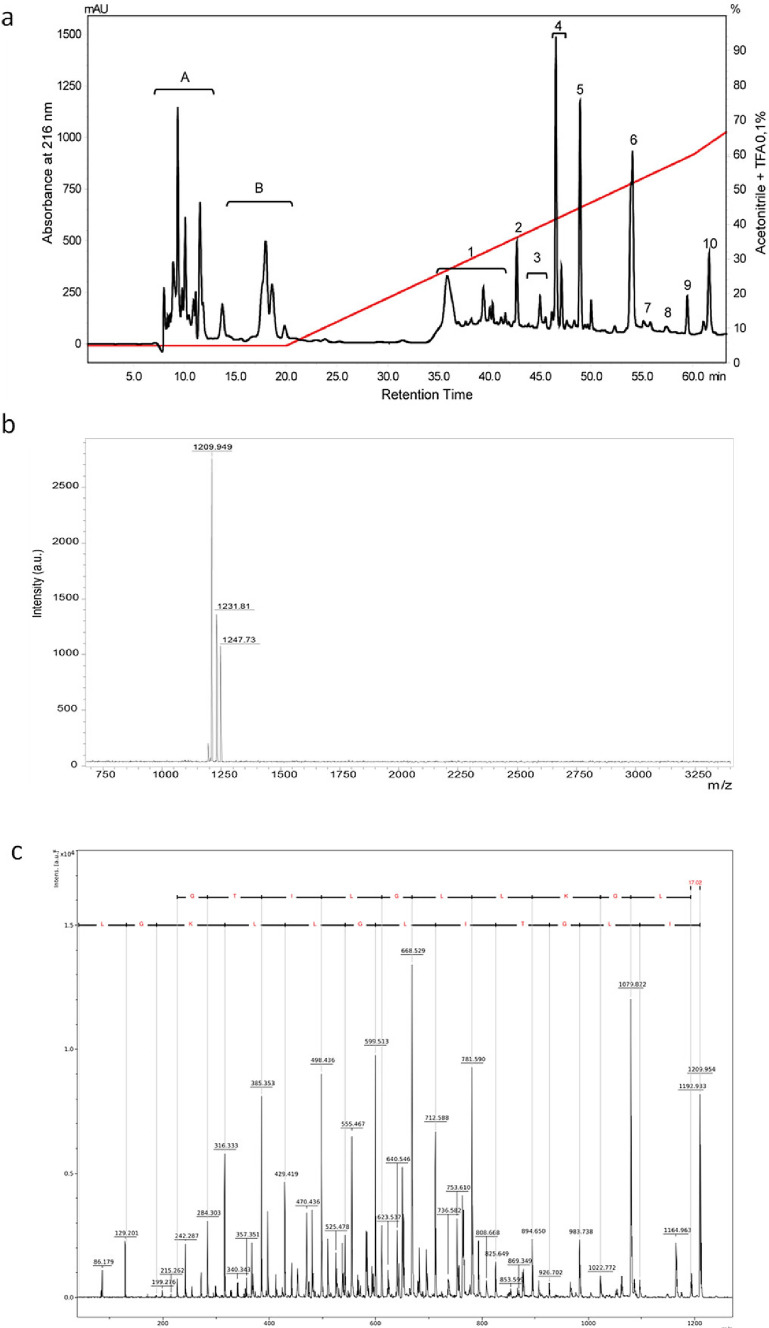
(**a**) High performance liquid chromatography fractionation (HPLC) of low molecular weight compounds found in the venom of *Parachartergus fraternus* social wasp. Ten fractions numbered 1–10 were collected for this work. (**b**) MALDI-TOF mass spectra of HPLC fraction 9, revealing the presence of ions [M + H]^+^  = 1209.94, [M + Na]^+^  = 1231.21 and [M + K]^+^  = 1247.79. (**C**) MALDI-TOF/TOF mass spectra in LIFT mode of the compound was obtained from fraction 9. Molecular mass analysis revealed a ten amino acid residues-long peptide with seven leucine/isoleucine ambiguities.

Fractionation of LMWC on a C18 reverse-phase column resulted in 10 different peptide fractions (Fig. 1A). The fractions indicated by the letters A and B correspond to biogenic amines and neurotransmitters such as γ-aminobutyric acid and glutamate. The MALDI-TOF mass spectrum of fraction 9 revealed the presence of a single compound, m/z 1209.949 (M + H^+^), indicating that the fraction contains a peptide of high purity (Fig. 1B).

The amino acid sequence was only partially determined by de novo sequencing of MS/MS spectrum (Fig. 1C) due to the presence of ambiguities, notably between isoleucine/leucine and lysine/glutamine amino acid residues. The corresponding peptide sequence was determined as I/L-I/L-G-T-I/L-I/L-G-I/L-I/L-Q/K-G-I/L-NH_2_. According to the spectrum, a reduction of about 17 Da was observed between the signal m/z 1209.9 and 1192.9, indicating the amidation of the C-terminal region.

### cDNA Library construction and sequence alignment

**Figure 2:**
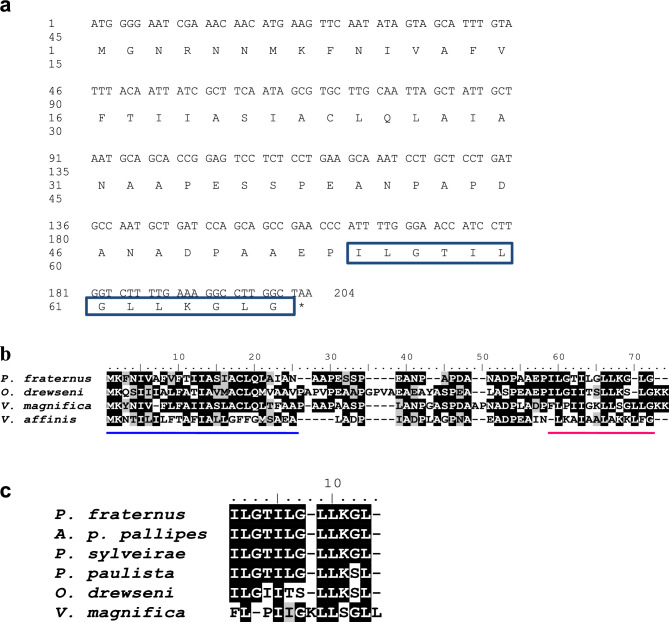
(**a**) The nucleotide sequences of precursors encoding Protonectin. (**b**) ClustalW sequence alignment of precursor peptides found in four wasp species: *Parachartergus fraternus*, *Orancistrocerus drewseni*, *Vespa magnifica* and *Vespa affinis*. represented above the blue line while mature peptides are represented above the red line. (**c**) ClustalW sequence alignment of mature peptides found in six wasp species: *Parachartergus fraternus* (*P. fraternus*), *Agelaia pallipes pallipes* (*A. pallipes pallipes*), *Protonectarina sylveirae* (*P. sylveirae*), *Polybia paulista* (*P. paulista*), *Orancistrocerus drewseni* (*O. drewseni*) and *Vespa magnifica* (*V. magnifica*).

In parallel with the MS profiling, we constructed a cDNA library of the venom gland, randomly sequencing twenty clones from this library (Fig. 2). One of the sequenced clones showed high similarity with peptide precursors identified in *Vespa magnifica* and *Orancistrocerus drewseni*^13,14^. The complete nucleotide sequence encoding protonectin was shown in the Fig. 2A. The sequence of this clone comprises 180 bp, and its in silico translation resulted in a putative peptide of 61 amino acid residues (Fig. 2B).

Computational analysis of this sequence by SignalP 4.1 software showed a cleavage signal between residues 26 and 27, suggesting the presence of a signal peptide. Alignment of the *P. fraternus* precursor with precursors of other peptides identified from *V. magnifica* and *O. drewseni* shows a high degree of identity (> 75%) in the signal peptide region, and in the propeptide sequence. Inclusion of the mastoparan precursor from *V. affinis* in the alignment shows that this region is less conserved when compared to the sequences of other wasp peptides, but the cleavage region is identical, suggesting that these two precursors are cleaved by similar mechanisms. Furthermore, the presence of a Glycine as the last residue before the stop codon is observed, which demonstrates that amidation can occur by post-translational processes.

The terminal precursor region identified in *P. fraternus* showed a mature peptide of 13 amino acid residues with the amino acid sequence I-L-G-T-I-L-G-L-L-K-G-L-G. The region corresponding to the mature peptides is also well conserved among protonectins and less conserved with the mastoparan sequence. Alignment of mature protonectin peptides isolated from different species shows the high degree of conservation of this peptide (Fig. 2C). The sequence of protonectin identified in *P. fraternus* is identical to that identified in *Protonectarina sylveirae*, which gives its name to this group of peptides, and in the venom of *Agelaia pallipes pallipes*^15^. This peptide is characterized by its high hydrophobicity, with 10 of the 12 amino acid residues having a hydrophobic nature. The amino acid at position 4 (threonine) is polar, with neutral charge, while the residue at position 10 (lysine) is positively charged and polar. Peptides isolated from *Polybia paulista*^16^ and *Orancistrocerus drewseni*^17^ have a substitution of glycine at position 11 for a serine (neutral polar), while the peptide from *O. drewseni* has another substitution at position 4, of threonine for a hydrophobic isoleucine.

### Design and synthesis of modified peptide

Little is known about the effects of the substitutions described above on the structure and activity of protonectin, and no data have been reported on its neuroactivity. To that end, modifications in the protonectin sequence of *P. fraternus* were designed for the chemical synthesis of a modified protonectin. The leucine residues at positions 2 and 8 were replaced by a phenylalanine residue, an amino acid of hydrophobic nature with an aromatic ring. The amino acid sequence of this novel peptide is IFGTILGFLKGL, amidated at the C-terminus. This peptide will be referred to as protonectin-F throughout this paper.

### Secondary structure characterization

Protonectin-F was characterized by Attenuated Total Reflectance-Fourier-Transform Infrared (ATR-FTIR; Supplementary Table S1) spectroscopy and by circular dichroism (CD; Supplementary Figure S2) and, in comparison to protonectin in aqueous media and in the presence of anisotropic environments, which are mimetics of membrane environments or the interactions with other proteins.

CD spectra of peptides in an aqueous environment suggest an unordered structure with a minimum around 198 nm, while in the presence of 40% TFE or of 8 mM SDS solutions a marked conformational change is observed. These spectra exhibit the features of helical conformations with double minima around 207 and 222 nm. The spectra also indicate that protonectin-F exhibits no preferential interaction for the anionic environment of the SDS solution over the electrically neutral TFE, while protonectin exhibits a weaker interaction with the anionic environment. These structural features were also observed by FTIR.

The presence of TFE or SDS solutions induces mostly α-helical and β-sheet structures as obtained from the deconvolution of CD spectra, which practically do not discriminate these peptides. Nevertheless, the deconvolution of the FTIR spectra indicates the absence of β-sheet in both peptides. In the case of protonectin-F, increased content of helices and decreased aggregation contributions were found, the latter representing an important feature for maximizing drug bioavailability. Both peptides exhibit similar strong amphipathicity according to the hydrophobic moment plot^18^; nonetheless, the lower level of the random coil in protonectin-F is also indicative of higher amphipathicity.

### Bioassays

#### Thermal pain induction model – hotplate

**Figure 3:**
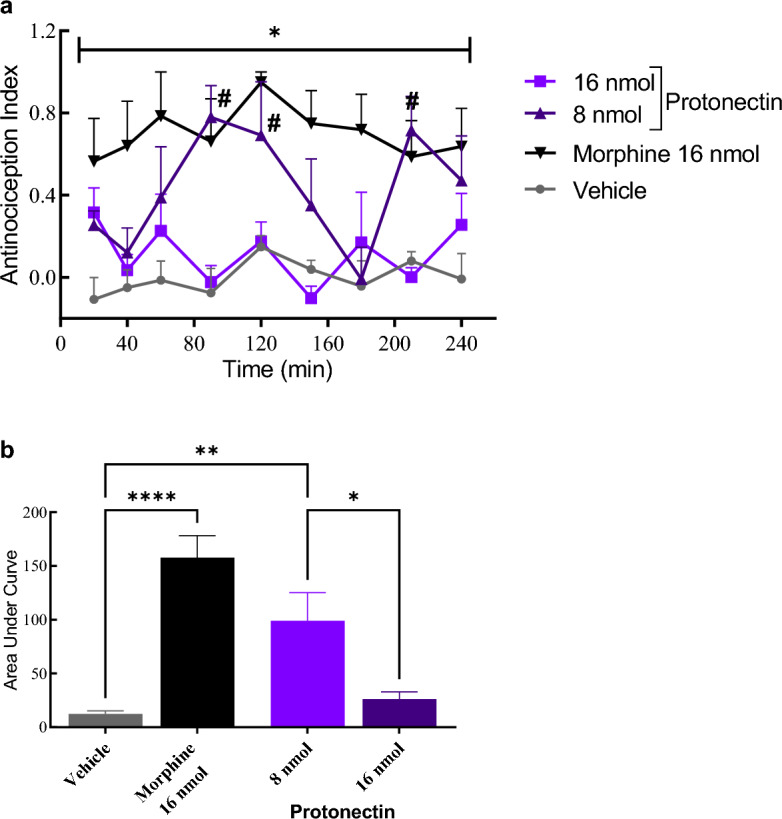
(**a**) Antinociception index obtained from the hot plate assay after i.c.v. injection of natural protonectin at 8 nmol/animal or at 16 nmol/animal. Control groups received either morphine at 16 nmol per animal or vehicle solution. Data were analyzed with Two-Way ANOVA followed by Bonferroni post-hoc test. (*) indicates statistical difference when compared morphine to vehicle control (* = *p* < 0.05). (#) indicates difference when compared Protonectin-F 16 nmol to vehicle control with *p* < 0.05. (**b**)- Area under curve obtained from the antinociception index results. Data were analyzed by ANOVA followed by Tukey’s post-hoc test. (*) indicates statistical difference (**** = *p* < 0.0001; ** = *p* < 0.01; * = *p* < 0.05).

Intracerebroventricular (i.c.v) injection of protonectin at 8 nmol/animal produced an antinociceptive effect 90, 120 and 210 min after injection (Fig. 3A). In all timepoints, the Antinociception Index (AI) of morphine was significantly greater than the vehicle (*p* < 0.05). On the other hand, administration of protonectin at 16 nmol/animal did not induced antinociceptive effect. Significant differences after analysis by Two-way Anova followed by Tukey as post-test were observed with regard to treatment [F_(3,15)_ = 14.92, *p* < 0.0001], but no differences were observed with respect to time [F_(3.481,52.21)_ = 1.965, *p* = 0.1221] and time versus treatment [F_(24,120)_ = 1.450, p = 0.0989]. An area under curve (AUC) analysis of the results (Fig. 3B) indicated significant differences between protonectin 8 nmol and the morphine control in relation to vehicle control [F_(3,15)_ = 19.93, *p* < 0.0001]. Moreover, significant difference was observed between both doses of the protonectin (*p* < 0.05).

**Figure 4:**
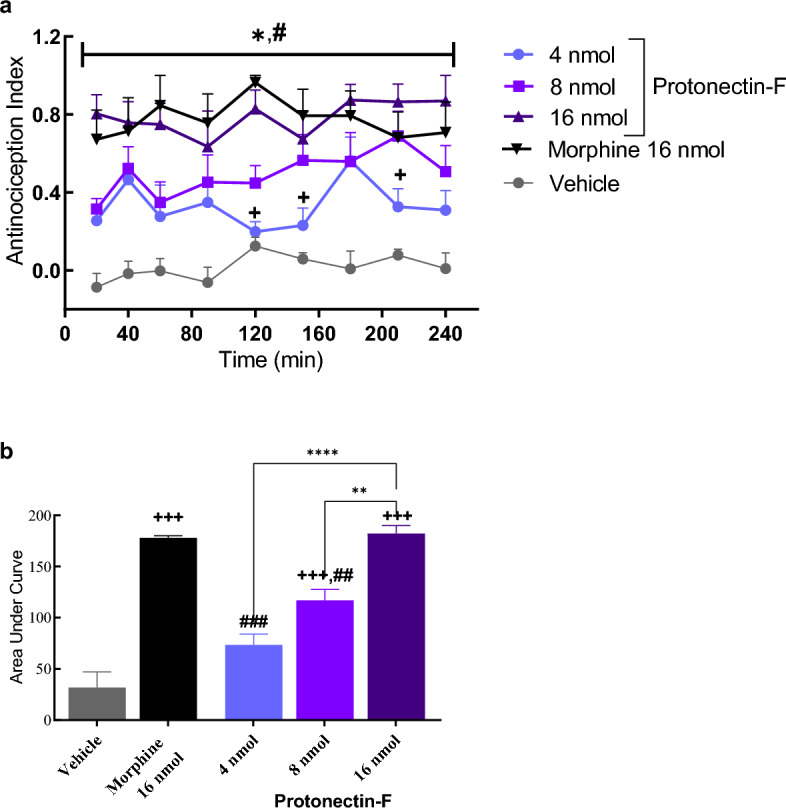
(**a**) Antinociception index obtained from the hot plate assay after i.c.v. injection of the modified protonectin-F at 16, 8 or 4 nmol/animal. Control groups received either morphine at 16 nmol per animal or vehicle solution. Data were analyzed with Two-Way ANOVA followed by Bonferroni post-hoc test. (*) indicates statistical difference when compared morphine to vehicle control. (#) indicates difference when compared protonectin-F 16 nmol to vehicle control with *p* < 0.05. ( +) indicates difference when compared to the group treated with protonectin-F at 16 nmol/animal with *p* < 0.05. (**b**) Area under curve obtained from the antinociception index results. Data were analyzed by ANOVA followed by Tukey’s post-hoc test. ( +) indicates statistical difference when compared to vehicle control (^**+++**^  = *p* < 0.001). (#) indicates difference when compared to morphine control with p < 0.05 (### = *p* < 0.001; ## = *p* < 0.01). (*) indicates difference when compared to the group treated with protonectin-F at 16 nmol/animal (**** = *p* < 0.0001; ** = *p* < 0.01).

Differentially, the bioinspirated peptide Protonectin-F demonstrated a potent dose-dependent antinociceptive effect, when injected by i.c.v. Significant differences were observed with regard to treatment [F_(4,18)_ = 29.21, *p* < 0.0001], but no differences were observed with respect to time [F_(8,18)_ = 1.48; *p* < 0.17] and time versus treatment [F_(32,144)_ = 0.598, *p* = 0.95]. Further analysis showed significant differences (p < 0.0001) between vehicle and protonectin-F in the 16 nmol/animal dose at all timepoints tested. When protonectin-F at 16 nmol/animal dose was compared to morphine, no significant difference was detected (*p* > 0.05, Fig. 4A). These differences were confirmed by the AUC (ANOVA followed by Tukey's post-test), in which a dose-dependent antinociceptive effect was observed (Fig. 4B) [F_(4,15)_ = 41.01, *p* < 0.0001]. The effects induced by the 16 nmol/animal of protonectin-F and morphine (16 nmol/animal) were not significantly different, while significant differences were observed for 8 nmol and 4 nmol/animal doses, as well as the vehicle when compared to morphine (16 nmol/animal) and protonectin-F 16 nmol/animal. These data show that at 16 nmol/animal, protonectin-F had an antinociceptive effect comparable to morphine at same dose.

### Motor performance assay

To evaluate motor deficits induced by the treatments, a single i.c.v. dose of the protonectin (16 or 8 nmol/animal), protonectin-F (16 or 8 nmol/animal) or morphine (16 nmol/animal) were administered to the animals (Supplementary Figure S3). A percentage of animals that failed the trial was calculated for each dose, Chi-square test indicated a significantly higher percentage of failure for animals treated with protonectin (60% failure) when compared to protonectin-F treated animals (10% failure) and vehicle (0% failure). Morphine at 16 nmol/animal dose induced motor impairment in 50% do animals, showing significant differences in relation to vehicle group (*p* < 0.05).

### In vitro immunomodulation assay

Protonectin-F was screened to assess potential cytotoxicity or hemolytic activity as previously described using a 3% suspension of fresh human red blood cells (Guilhelmelli et al., 2016). The results showed that protonectin-F presented both activities only at high concentrations of 50 µM and 100 µM, suggesting low overall cell toxicity (Supplementary Figure S4).

**Figure 5:**
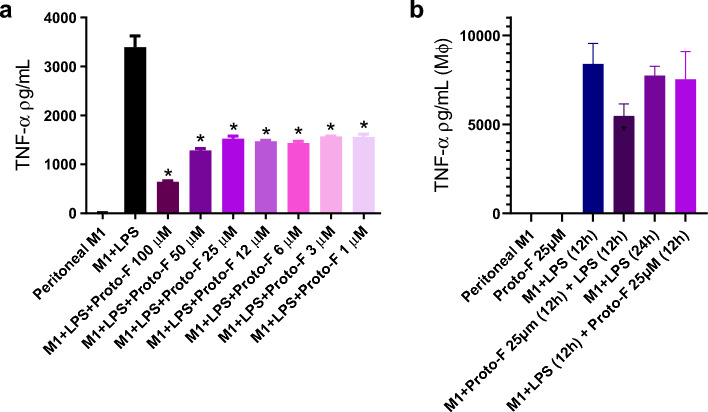
(**a**) TNF-α production after LPS insult. Every concentration of protonectin-F tested was able to reduce TNF-α production of peritoneal macrophages. Data were analyzed by One-Way ANOVA followed by Tukey’s post-hoc test. Statistically significant differences are indicated when compared to M1 + LPS control (*). (**b**) Demonstration of time dependence of protonectin-F immunomodulation. Data were analyzed by One-Way ANOVA followed by Tukey’s post-hoc test. Statistically significant differences are indicated when compared to M1 + LPS (12 h) control (*).

The capability of the peptide to modulate TNF-α production was verified in an in vitro assay, where murine peritoneal macrophages interacted with the peptide (1.56–100 μM) following LPS stimuli, and it was shown that this treatment reduced TNF-α, demonstrating a possible anti-inflammatory activity. An in vitro assay was also performed to evaluate if protonectin-F reduces TNF-α production either before or after LPS stimuli. At a concentration of 25 μM the peptide reduced the production of the pro-inflammatory cytokine when it interacted with the peritoneal macrophages before LPS stimuli (Fig. 5).

### Pharmacological interaction with naloxone and AM251

**Figure 6:**
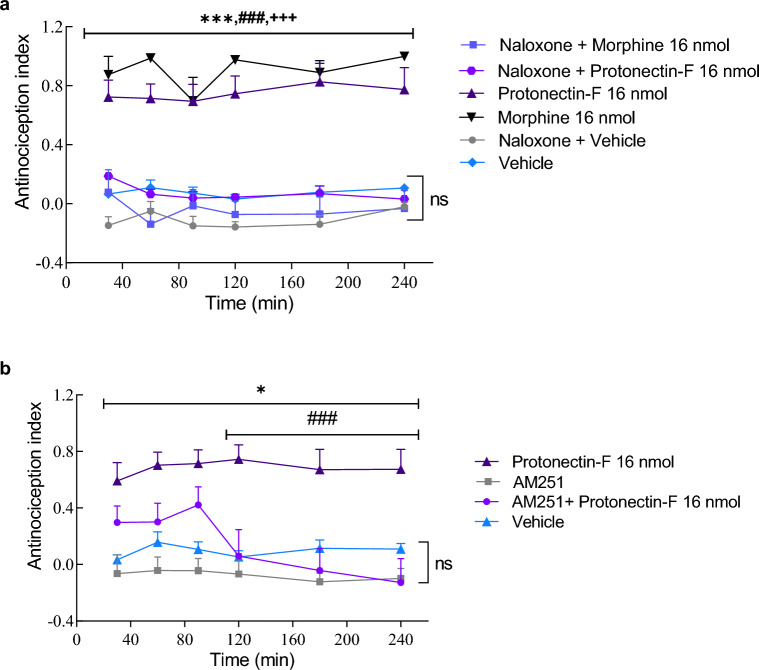
(**A**) Antinociception index obtained from the hot plate assay exploring the pharmacological antagonism of naloxone. The antinociceptive effect of protonectin-F (16 nmol) and morphine (16 nmol) was evaluated after administration of naloxone, as well as protonectin-F or morphine alone. Control groups were treated with vehicle alone or with naloxone and vehicle. Data were analyzed by Two-Way ANOVA followed by Bonferroni’s post-hoc test. (***) indicates statistical difference when compared to vehicle control *p* < 0.001. (###) indicates difference when compared the group treated with naloxone and morphine 16 nmol with *p* < 0.001. (+ + +) indicates difference when compared to the control group treated with naloxone and protonectin- F with *p* < 0.001. (**B**) Antinociception index obtained from the hot plate assay exploring the pharmacological antagonism of AM251. The antinociceptive effect of protonectin-F was evaluated after administration of AM251, as well as protonectin-F alone. Control groups were treated with vehicle or with AM251. Data were analyzed by Two-Way ANOVA followed by Tukey’s post-hoc test. (***) indicates statistical difference when compared to vehicle control with *p* < 0.001. (###) indicates difference when compared the group treated with AM251 and protonectin-F with *p* < 0.001.

Treatment with morphine (16 nmol/animal) induced a high antinociception index (AI) for all timepoints observed (*p* < 0.05) when compared to the vehicle treated group, while treatment with both morphine and opioid antagonist naloxone induced no differences in AI when compared to vehicle control (Fig. 6A). Multivariate analysis of variance (MANOVA) revealed significant difference of the treatment (F_(5,48)_ = 29.36; *p* < 0.0001) and of the interaction between treatment and time (F_(5,240)_ = 0.2995; *p* = 0.9128). Hence, administration of naloxone abolished the antinociceptive effect of morphine. Similar results were observed for the protonectin-F (16 nmol/animal) treated group. While protonectin-F treated animals exhibited a higher AI than vehicle treated groups (*p* < 0.05) throughout the whole experiment, the administration of both protonectin-F and naloxone abolished that difference, indicating a potential activator of the descending pain pathway for protonectin-F.

A similar experiment was performed using the cannabinoid antagonist AM251 (Fig. 6B). While animals treated with protonectin-F presented a higher AI when compared to the vehicle group (*p* < 0.05) at all timepoints observed, the treatment with both protonectin-F and AM251 also resulted in an AI no different from that of the vehicle control group in the experiment starting from 120 min [MANOVA: treatment F_(3,27)_ = 24.71, *p* < 0.0001; time F_(3.821,103.2)_ = 1.530, *p* = 0.2013; and treatment versus time F_(15,135)_ = 1.100, *p* = 0.3621].

### Tolerance assay

**Figure 7:**
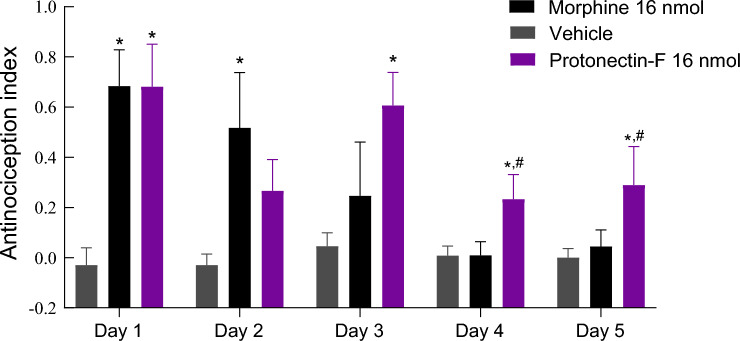
Antinociception index obtained from the hot plate assay after i.c.v injection of Protonectin-F (16 nmol) or morphine (16 nmol). Control group was treated with vehicle alone. All experimental groups were treated for five consecutive days and the antinociception was measured 120 min after injection. Data were analyzed by Two-Way ANOVA followed by Tukey’s post-hoc test. (*) statistically significant differences (*p* < 0.05) are indicated when compared to vehicle control, (#) statistically significant differences (*p* < 0.05) are indicated when compared to morphine group.

As expected, the continuous treatment with morphine (16 nmol/animal) resulted in a reduction of its effect (Fig. 7). Repeated measures of analysis of variance (RM-ANOVA) revealed significant differences between treatments (F_(2.804,39.25)_ = 5.320, *p* = 0.0042), time (F_(2,16)_ = 11.57, *p* = 0.0008) and the interaction between treatment and time (F_(8,56)_ = 2.609, p = 0.0168). While on days 1 and 2 the animals treated with morphine presented an AI significantly different from vehicle treated animals (*p* < 0.05), no difference was observed between morphine and vehicle groups at days 3, 4 and 5. Regarding the animals treated with protonectin-F (16 nmol/animal), significant differences compared to vehicle treated animals were observed at days 1, 3, 4, and 5. These results indicated a lesser tolerance effect of protonectin-F compared to the morphine treatment.

### FLIPR Ca^*2*+^ assays

High concentrations of the peptides (> 10 µM) can elicit a rapid and intense Ca^2+^ influx in the SH-SY5Y cells (supplementary Figure S5). Interestingly, most cells returned to basal intracellular Ca^2+^, but they were not responsive to further stimuli. Thus, the peptide-evoked Ca^2+^ overload might cause cell damage and, surprisingly, protonectin-F was more toxic than protonectin (EC_50_: 21.51 ± 0.43 µM vs. 57.87 ± 1.33 µM, respectively).

At peptide concentrations that did not elicit Ca^2+^ responses, we evaluated how protonectin and protonectin-F pre-treatment may affect the Veratridine and Nicotine-evoked Ca^2+^ responses. Veratridine activates voltage-gated Na^+^ channels expressed in SH-SY5Y cells, causing an inward Na^+^ current that leads to membrane depolarization. The depolarization activates voltage gated Ca^2+^ channels, which increase intracellular Ca^2+^ concentrations. Veratridine-evoked Ca^2+^ responses (Supplementary Figure S5) were correlated to neither protonectin (Pearson r = 0.3275, *p* = 0.3557) nor protonectin-F (Pearson r = 0.2158, *p* = 0.5493) treatment. Also, Nicotine activates the ligand-gated nAChR channels endogenously expressed in SH-SY5Y cells. Neither of the peptides, protonectin (Pearson r = -0.4821, *p* = 0.2324) and protonectin-F (Pearson r = -0.6037, *p* = 0.3645), affected the Nicotine-evoked Ca^2+^ responses (Supplementary Figure S5).

Furthermore, protonectin-F does not present activity in the μ and κ opioid receptors (Supplementary Figure S6) or on the acid-sensing ion channels (ASICs) rat ASIC1a and ASIC1b, human ASIC1a, and rat ASIC2a (Supplementary Figure S7).

## Discussion and conclusions

The treatment of chronic pain is a fundamental human right, and the Montreal Declaration, based on the principles of the World Health Organization (WHO), states that all those suffering from pain have guaranteed their right to access pain treatment ^19^. Conventional therapies and the use of systemic analgesics are widely indicated in chronic pain conditions. However, it is estimated that 30–50% of patients with neuropathic pain do not present significant clinical improvement. Moreover, opioid drug-induced adverse effects are major complications in therapy^20,21^. In view of these limitations, animal venom represents a rich source of novel molecular templates for the development of pharmaceuticals such as analgesics, considering the wide range of bioactive molecules it possesses. Peptides derived from animal venom often exhibit high affinity, binding specificity, and ample potency profiles due to evolutionary pressures^22^ and many of them act on targets associated with pain modulation, such as ion channels and membrane receptors^23^.

Based on these promising characteristics, in the present work, we investigated the low molecular weight components of the venom of the *P. fraternus* wasp, coupling transcriptomic and proteomic strategies to identify a 12-amino-acid-long peptide called protonectin. Previous studies from our group identified other neuroactive peptides in the venom of *P. fraternus*. The mastoparan Agelaia-MP-I was identified in the venom and revealed an antinociceptive effect when administrated via i.c.v^24^. A second peptide called fraternin demonstrated neuroprotective activity in a mouse model of parkinsonism^25^. The sequence of the protonectin from *P. fraternus* is identical to protonectins identified in two other wasp species, *A. pallipes pallipes* and *O. drewseni*. This peptide was characterized as mast cell degranulating, antibiotic, antifungal, and chemotactic, and more recently a potential anticancer effect was described^26^. Yet, this is the first work to characterize the potential neuroactive activity of protonectin in a thermal-induced model of nociception.

Our results showed that the administration of 8 nmol/animal of protonectin induced an antinociceptive effect; however, when the dose was increased to 16 nmol/animal, the effect was reduced. The higher dose also induced a high failure rate in a motor performance assay, indicating that this peptide can induce some sort of toxicity interfering in the nociception assay. In an attempt to counteract this outcome, we substituted the leucine residue at positions 2 and 8 by the phenylalanine residue, resulting in a novel peptide called protonectin-F. These modifications increased the α-helix contribution in its secondary structure and decreased the aggregation contribution, modifications that might increase protonectin-F bioavailability. Protonectin-F not only maintained the antinociceptive effect comparable to that of morphine but also demonstrated a dose-dependent activity.

Considering the relevance of opioid and cannabinoid pathways in pain modulation, we further evaluated the antinociceptive effect of protonectin-F in the presence of either the opioid antagonist naloxone or the cannabinoid antagonist AM251. In both instances, the effect of protonectin-F was abolished. Considering these results and the intense colocalization between cannabinoid and opioid receptors, we could not discriminate if protonectin-F presents any affinity for either pathway. Nonetheless, protonectin-F is able to activate both pathways, either directly or indirectly. The assays performed in vitro to characterize the mechanism of action of protonectin-F indicated that it lacks activity in any of the receptors evaluated, suggesting that this peptide possibly acts in activation of the pain descending pathway by other receptors. This infers that protonectin-F may be a non-promiscuous antinociceptive peptide and more assays are necessary to identify the exact mechanism of action.

Chronic exposure to morphine can paradoxically exacerbate and prolong pain hypersensitivity, changing pain from acute to chronic. This event is called the “two-hit” hypothesis, wherein morphine exacerbates the pro-inflammatory response after injury^27^. We observed that daily administration of protonectin-F did not induce a tolerance effect as intense as the one seen with the administration of morphine. This result is especially interesting, considering that tolerance and the resulting need for increased doses for the same effect is a major disadvantage of the continuous use of morphine for the treatment of chronic pain.

Considering the possibility that antinociceptive compounds modulate inflammation, we tested protonectin-F in both in vitro and in vivo assays. The results showed a reduction of TNF-a release in vitro in all concentrations tested, an effect possibly due to LPS activity blockage. Likewise, other new substances have shown better analgesia than morphine, such as ZH853, which also reduces the inflammatory response versus morphine and vehicle^28^, bringing hope to the discovery of potent analgesics that do not involve the tolerance effect setbacks.

The association between pain and an immune response is well established. Several products secreted during the immune response activation can modulate chronic pain, such as the release of cytokines (IL-8, IL-1b, IL-6, TNF-a), chemokines, pro-excitatory lipids (like prostaglandins), and damage-associated molecular patterns (DAMPS) (reviewed in^29^). One Toll-like receptor described associated with pain is TLR-4 in the arthritis model, and the use of TLR-4 antagonists during the early phases of inflammation affected the transition from acute to chronic pain^30^. Our results showed that treatment with the protonectin-F before the LPS stimulation reduced the release of TNF-a, probably collaborating with the reduction seen in tolerance effects. In this context, protonectin-F can be able to act as an analgesic in important phases of pain, reducing the peripheral inflammation and decreasing the establishment of chronic pain.

In conclusion, given their attractive pharmacological profile, venom compounds represent an invaluable source of molecules for the design of novel therapeutics intended for the future treatment of neurological and immune disorders. As observed herein, strategies that involve the use of rational peptide design represent an important tool to improve chemical and physical properties in natural peptides, thus, granting enhanced qualities to new peptides. The peptide identified in this study showed a diversity of biological effects, ranging from antinociception to lung tissue protection. Relevantly, the antinociceptive effect observed was comparable to that of morphine while not inducing tolerance. Additional biological characterization might reveal new effects of protonectin-F, furthering its potential as a pharmacological tool.

## Methods

### Biological material and venom extraction

After proper licensing (license ICMBio number 21723–2/process CNPq number 010476/2013–0), nests with specimens of the social wasp *Parachartergus fraternus* were collected at the Darcy Ribeiro Campus of the University of Brasília, Federal District, Brazil. The identification of the species was kindly performed by Prof. Dr. Fernando B. Noll from the UNESP Department of Zoology and Botany (Brazil).

Specimens (~ 2,500 individuals) were immediately frozen at − 20 °C for 24 h for euthanasia, and then the crude venom from glands and reservoirs (2,380 gland sacs) was extracted. Thereafter, all material was macerated, homogenized in 1:1 acetonitrile and deionized water, and centrifuged at 10,000 g for 3 min at 4 °C. Then, to obtain the crude venom, the supernatant was removed, frozen, and dried under vacuum. Finally, the crude venom was solubilized in 1:1 acetonitrile and deionized water and ultrafiltered in a Microcon filter (Millipore®) thus obtaining compounds of molecular mass < 3 kDa, known as low molecular weight compounds (LMWC). These compounds were resuspended in deionized water and acetonitrile (5%) for the isolation step.

### Venom purification and identification

The fractionation of the LMWC was performed using reverse-phase high-performance liquid chromatography (HPLC) in the Shimadzu® Prominence system with a C18 column (C18 ODS, 15 µm, 250 × 10 mm, Phenomenex®, Torrance, CA, USA). The procedures were performed following the protocols described by^25^. Deionized water with 0.1% trifluoroacetic acid (TFA) was used as solvent A, while acetonitrile with 0.1% TFA was used as solvent B. The sample was eluted at 5% B gradient for 20 min and then 5–60% B for 40 min, with absorbance monitored at 216 and 280 nm. Each fraction was manually collected and vacuum dried in a SpeedVac Concentrator (Thermo Scientific®, USA).

For mass identification, chromatographic fractions were subjected to Ultraflex III MALDI-TOF/TOF mass spectrometer (Brucker Daltonics®, Germany). The fraction of interest was mixed with deionized water and then added to an α-cyano-4-hydroxycinnamic acid matrix. Later, this mixture was deposited in triplicate on a Bruker MTP Massive plate and, once crystallization was attained, samples were ready for analysis. To achieve greater accuracy in obtaining the varied mass spectra (MS), equipment was operated in a positive reflector mode. For the MS/MS analysis, we used the LIFT method aiming sequence elucidation. The de novo sequencing and the interpretation of the obtained spectra were performed manually with the aid of FlexAnalysis 3.0 software (Brucker Daltonics®, Germany).

### Venom cDNA library construction

We extracted total RNA from *P. fraternus* specimens (*N* = 20) using the ZR-Duet™ DNA / RNA Miniprep kit (Zymo Research, USA), according to the manufacturer's instructions. Then, a PCR-based cDNA library was constructed following the specifications for the SMARTer® PCR cDNA Synthesis kit (Takara Bio USA Inc., USA). The inserts obtained were linked to a linearized pSMART2IF vector (Takara Bio USA Inc., USA) and transfected into competent *Escherichia coli* DH5-α for cDNA amplification. Plasmid DNA was extracted and purified from the clones and taken to the sequencing platform (Model 3100, Applied Biosystems, Foster City, CA).

Bioinformatics analyses were performed as previously described in^31^. In brief, to extract the high-quality sequence region, the sequences were subjected to the Phred program with the window length set to 100 and the standard quality to 20. The CrossMatch program was used to remove vector sequences. ESTs that shared the identity of 95 out of 100 nucleotides were assembled in contiguous sequences with the CAP3 program. All these bioinformatics analyses were simultaneously run at the http://www.biomol.unb.br/ site using the default setup. The cDNA sequences were searched against a public database using blastx and blastn algorithms to identify putative functions of the new ESTs. The theoretical molecular masses of the putative mature peptides were calculated in the online service PeptideMass http://www.expasy.ch/tools/peptidemass.html.

### Peptide alignment and comparison with databases

After obtaining the peptide sequences of interest, searches were made for similarities with peptides previously isolated from the venom of Hymenoptera. For this, appropriate databases were used, which corresponded to Uniprot (available at http://www.uniprot.org/) and BLASTP (available at < http://blast.ncbi.nlm.nih.gov). Once similarities were found, the peptide sequences were aligned using the Clustal Omega software (available at < http://www.ebi.ac.uk/Tools/msa/clustalo/ >).

### Solid-phase peptide synthesis

The protonectin and protonectin-F sequences were determined to be ILGTILGLLKGL-NH_2_ and IFGTILGFLKGL- NH_2_, respectively. Peptides were synthesized through standard FMOC chemistry (FastBio, Brazil), and samples were inspected by mass spectrometry to certify the peptide’s monoisotopic mass, purity, and amino acid sequence. The long-term stability was also monitored every 6 months, using the same parameters applied for the natural compound.

### Circular dichroism (CD) spectroscopy

Peptide samples were prepared by diluting the stock solution in ultra-pure water (or in D_2_O for ATR-FTIR experiments) containing either 40% 2,2,2-trifluoroethanol (2,2,2-trifluoroethanol-d3, TFE), or 8 mM sodium dodecyl sulfate (SDS) For ATR-FTIR experiments, samples were left incubating for 2 h prior to the acquisition of spectra.

CD spectra were acquired in the absence of peptides and their presence at 20 μM concentration, at 25 °C. A 0.5 cm path length cell was used to record spectra from 260 to 193 nm with a Jasco-815 spectropolarimeter (JASCO International Co. Ltd., Tokyo, Japan), at a scan speed of 50 nm/min, bandwidth of 1.0 nm, 0.5 s response, and 0.2 nm resolution. Final spectra represent the average of over ten scans. Baseline correction was applied. The mean-residue ellipticity, [Θ] (deg cm2/dmol), was calculated with the relationship [Θ] = 100θ/(lcn), where θ (mdeg), is the observed ellipticity, ‘l’ the path length in cm, ‘c’ (mM) the peptide concentration, and ‘n’ the number of peptide bonds. Spectra were fitted with the software CD Pro (configured with CONTIN/LL and using the reference set SMP56).

### Attenuated total reflectance-fourier-transform infrared spectroscopy (ATR-FTIR)

FTIR spectra were recorded in the absence and the presence of peptides at 1 mM concentration, from 1600 to 1700 cm^-1^ with Spectrum Two spectrophotometer (Perkin Elmer, Beaconsfield, UK), fitted with an ATR device. Aliquots of 7 μL of each sample were deposited on top of the ATR crystal device. For each spectrum, 20 scans were collected with a resolution of 2 cm^-1^. The background was the clean crystal. To assign the wavenumbers of the component bands in the spectra, baseline correction was applied, overlapping bands were resolved and the second-derivative spectra were smoothed by 13-data point Savitsky-Golay. The ATR-FTIR spectra were analyzed to give the estimates of the relative contents of the secondary structure elements included in the whole area of the amide I band, based on the fraction of the total area under the peak. The deconvolution of the amide I region considered the following wavenumber ranges and assignments for the secondary structure elements: from 1620 to 1625 cm^-1^, aggregated β-sheet; 1625–1640 cm^-1^ β-sheet, from 1645 to 1655 cm^-1^, α-helix; 1656–1670 cm^-1^, distorted helix; 1670–1680 cm^-1^, turns^32,33^.

### Bioassays

#### Animals

Female Swiss mice and male and female BALB/c mice aged 6–12 weeks were obtained from the Animal Facility of the Institute of Biological Sciences at the University of Brasilia (Federal District, Brazil). Animals were housed six per cage under a light/dark cycle of 12 h with controlled temperature (25 °C) and humidity (55%). Throughout the experimental period, food and water were offered ad libitum. Mice were randomly assigned to experimental groups. All animal experiments were performed in accordance with NIH and ARRIVE guidelines, and were properly approved by the Animal Ethics Committee of the University of Brasília (UnBDoc 63,878/2011). All methods were carried out in accordance with relevant guidelines and regulations.

### Neurosurgery and treatment administration

For the i.c.v. administration of the treatments, mice were anesthetized intraperitoneally with ketamine (Dopalen, Ceva®, Brazil) and xylazine (Anasedan, Ceva®, Brazil) (75 and 15 mg/kg, respectively) diluted in saline (0.9% NaCl) and then placed in a stereotaxic frame (Insight Equipamentos®, Brazil). After proper cleaning and hair removal from the head, a local subcutaneous injection of lidocaine 2% (Lidostesim® 3%, Dentsply Pharmaceutical®, Brazil) was given, followed by skull exposure by a small incision in the scalp. A guide cannula (0.7 mm external diameter, 10.2 mm long) was implanted in the lateral cerebral ventricle at the following coordinates:—0.2 mm anterior to the bregma, 1.0 mm lateral to the midline, and—2.3 mm ventral to the skull’s surface, according to The Mouse Brain in Stereotaxic Coordinates ^34^. Once the implantation was completed, we applied a topical ointment (Neomycin and Bacitracin Sulfate—Nebacetin®) which aids healing and prevents local infections. All animals were housed in their cages after surgery, and after 4–6 days of recovery mice were submitted to behavioral bioassays.

A macroscopic analysis was required to evaluate if the cannulas were correctly positioned. Hence, at the end of each bioassay, animals were euthanized by thiopental overdose (120 mg/kg) and methylene blue was injected through the guide cannula, followed by brain removal and fixation with formalin 4% at 4 °C for 24 h. The correct positioning of the guide cannula and the injection site (ventricle stained in blue) were verified by performing manual cuts using an acrylic matrix (Insight Equipamentos Ltda®, São Paulo, Brazil). Only animals that had their cannula properly implanted were considered valid to proceed in our study.

Intracerebroventricular administration was performed by coupling a 10.1 mm long needle associated to a polyethylene tube into the guide cannula. With the aid of an infusion pump coupled to a precision syringe (Harvard Apparatus Compact Infusion Pump®, USA) we injected a final volume of 2 μL/animal at a speed of 1.60 μL/minute. Independent experimental animal groups (*N* = 4–6 per group for protonectin; *N* = 8–10 per group for protonectin-F) were treated with vehicle solution (deonized water), morphine (16 nmol), and peptides, all injected i.c.v.

### Thermal pain induction model-hotplate

Animals were tested in a random and blind manner for thermal withdrawal nociception using a hot plate apparatus (AVS Projetos®, Brazil), comprised of a metal plate maintained at 55 ± 0.5 °C enclosed with four acrylic walls to prevent the animal from escaping. Independent groups of animals (*N* = 6–8 per group) were treated either with vehicle, morphine sulfate (16 nmol/animal), protonectin-F (16, 8 and 4 nmol/animal) or protonectin (16 and 8 nmol/animal).

Mice were observed for signs of nociception, i.e. licking of the paws, jumping, and rigorous swinging of the hind paw. Response latencies were recorded and animals were removed from the plate immediately upon licking a hind paw or if no response occurred within 30 s to avoid tissue damage. Prior to each neurosurgery, animals were selected for thermal withdrawal nociception response. Animals were discarded from the test if the baseline response was less than 3 s or longer than 15 s.

At test day, animals were also pre-evaluated and only those with escape latency between 3 and 15 s were maintained and submitted to the assay. Before peptide administration, each animal was tested three times to determine a baseline response that resulted from the average of three escape latencies with 5 min intervals. Measured escape latencies were normalized to an antinociception index (AI) using the following formula:

$$AI= \frac{Escape\,latency-Baseline}{Maximum\,Latency-Baseline}$$

### Motor performance assay

To evaluate possible nonspecific effects of the peptides on motor coordination, mice were trained and tested on a rotarod apparatus (Insight Equipamentos Ltda®, São Paulo, Brazil) for a total of 4 days. For training purposes, mice were allowed for the first three experimental days to walk on the rod for 5 trials with a duration of 3 min per trial. On the fourth day, each mouse was submitted to only one trial of 3 min duration. Only animals that successfully completed this task were then randomly allocated to the experimental groups.

After the selection was completed, animals were treated i.c.v. with a single dose of morphine (16 nmol), protonectin (16 and 8 nmol) or protonectin-F (16, 8 and 4 nmol) and then were tested 10, 20, 40, 60, 90, 120, 150, 180, 210, and 240 min after peptide administration. The percentage of animals that failed to complete a 3-min trial was calculated for each dose.

### Pharmacological interaction with naloxone and AM251

To evaluate if the antinociceptive effect of protonectin-F was associated with opioid or cannabinoid pathways, a pharmacological interaction assay was performed similarly to the thermal pain induction model described above. For the opioid antagonist pharmacological interaction, we established the following treatment groups: vehicle group: treated with 0.9% saline intraperitonially and deionized water i.c.v., naloxone group: naloxone hydrochloride (4 mg/kg, intraperitonially) and deionized water i.c.v., morphine interaction group: naloxone hydrochloride (4 mg/kg, intraperitonially) and morphine sulfate (16 nmol/animal, i.c.v.) and protonectin-F interaction group: naloxone hydrochloride (4 mg/kg, intraperitonially) and synthetic peptide (16 nmol/animal, i.c.v.).

In the case of the pharmacological interaction assay with cannabinoid antagonist, we used protonectin-F (16 nmol/animal) and AM251 (13 nmol/animal). For the cannabinoid interaction, we established the following treatment groups: vehicle group: treated with deionized water i.c.v., AM251 group: AM251 (13 nmol, i.c.v.), and protonectin-F interaction group: AM251 (13 nmol, i.c.v.) and synthetic peptide (16 nmol/animal, i.c.v.).

### Tolerance assay

Either protonectin-F (16 nmol/animal), vehicle or morphine (16 nmol/animal) was administered i.c.v for 5 days to evaluate if the animals became tolerant to the treatment. Each day the animals were challenged in the hot plate test 120 min after the treatment, as this is the peak time of protonectin-F effect. The antinociception index was calculated for each animal as previously indicated.

### Calcium measurement

The assays were performed using a fluorescence imaging plate reader (FLIPR TETRA; Molecular Devices®) with the neuroblastoma cell line SH-SY5Y. SH-SY5Y endogenously expresses the voltage-gated channels of sodium (Na_v_ 1.2, 1.3, and 1.7), calcium (L and N-type), and ligand-gated nAChR channels (α3- and α7-containing)^35,36^. SH-SY5Y cells were cultured in RPMI containing 15% fetal bovine serum (FBS) and supplemented with L-glutamine. Cells were grown to 70–80%, plated at a density of 50,000 cells/well on black-walled 384-well imaging plates, and cultured in a humidified incubator set at 37 °C and 5% CO_2_ for 48 h. Next, the medium was removed, and SH- SY5Y cells were loaded with 20 µl/well Calcium 4 dye reconstituted in assay buffer ^36^. The fluorescence was read every second and expressed as a response over baseline. The peptide (100–0.024 µM), protonectin or protonectin-F, was added after 10 baseline reads. Then, after 5 min, cells were stimulated by the addition of Veratridine (50 µM) or Nicotine (30 µM), and fluorescence was recorded for a further 5 min.

### AlphaScreen cAMP assay – opioid receptors

In the opioid receptor assay, HEK 293 (RRID:CVCL_0045) cells were previously transfected with FLAG-KOP and FLAG-MOP ^37^, separately. The cells were grown in DMEM supplemented with 10% FBS and antibiotic G418 sulfate (0.5 mg mL^-1^) used only after confluence. The cells were harvested using Versene (Invitrogen) and resuspended in Hanks buffered balanced salt solution with bovine serum albumin (0.1%, BSA), 3-isobutyl-1-methylxanthine (0.5 mM,), and HEPES (5 mM) at pH 7.4. Cells were seeded at a concentration of 5 × 10^3^ cells per well for HEK-KOP cells and 2 × 10^4^ cells per well for HEK-MPO, in 96-well plates, The AlphaScreen cAMP kit (PerkinElmer, MA, USA) was used following the manufacturer’s instructions. Forskolin (100 μM) was added as an adenylyl cyclase stimulator, and the cells were incubated for 1 h with the peptides (1 pM—10 μM) or the agonists of KOP and MOP, u50488H, and fentanyl, respectively. After that, in the dark, streptavidin donor beads (1 unit/25 μL) and biotinylated cAMP (1 unit/25 μL) were added and incubated overnight^37,38^. The cAMP signal was visualized in a multiplate reader.

### Oocyte electrophysiology–ASICs channels

Peptide activity was assessed using two-electrode voltage-clamp (TEVC) experiments performed on *Xenopus laevis* oocytes expressing homomeric rat (r) and human (h) ASIC channels. *X. laevis* stage V-VI oocytes were removed and treated with collagenase (Sigma type I) for defolliculation. cRNA encoding ASICs was synthesized using an mMessage mMachine cRNA transcription kit, and oocytes were injected with 0.5–5 ng cRNA per cell. Oocytes were kept at 17 °C in ND96 solution (96 mM NaCl, 2 mM KCl, 1.8 mM CaCl2, 2 mM MgCl2, 5 mM HEPES; pH 7.45) supplemented with 5 mM pyruvic acid, 50 µg/mL gentamicin, 50 μg/mL streptomycin, and 2.5% fetal horse serum. Experiments were performed at room temperature (21–22 °C) 1–4 days after cRNA injection. HEPES was replaced by MES to buffer solutions at pH < 6.8. Oocytes were clamped at –60 mV (Axoclamp 900A; Molecular Devices, CA, USA) using microelectrodes filled with 3 M KCl solution (0.5–1.0 MΩ resistance). Data acquisition (sampled at 5 kHz and filtered at 0.01 Hz) was performed using pClamp10™ Software Suite (Molecular Devices, CA, USA). Currents were elicited by a drop in pH from 7.45 to 6.0 for rASIC1a/1b and hASIC1a, and pH 4.5 for rASIC2a every 60 s using a micro-perfusion system to allow rapid solution exchange. Protonectin-F was applied post-stimulation at pH 7.45 and oocytes were bathed in the toxin solution until the next stimulation (~ 50 s exposure). Solutions in the presence of peptide contained 0.05% fatty acid BSA-free to prevent adsorption to plastic ware and tubing. Currents were normalized to control currents without peptides^39^.

Animal work was carried out in strict accordance with the recommendations in the Australian code of practice for the care and use of animals for scientific purposes (8th Edition). Frog recovery surgery was performed under anesthesia (animals were bathed in 1.3 mg/mL MS-222), and all efforts were made to minimize suffering. The minimum time between surgeries on the same animal was three months, and on the final surgery (maximum of six months) frogs were euthanized by decapitation under MS-222 and ice anesthesia.

### Cytotoxicity assay and hemolysis assay

The experiments were conducted using BALB/c (*Mus musculus*) wild type mice, male and female, aged 6–12 weeks. The animals were kept with food and water ad libitum in the animal facility of the Institute of Biological Sciences (IB), University of Brasilia. The procedures were approved by the Animal Ethics Committee of the University of Brasília and conducted according to the Brazilian Council for the Control of Animal Experimentation (CONCEA) guidelines (UnBDoc n°. 46/2017).

After 3 days of the intraperitoneal stimuli with thioglycolate, peritoneal macrophages were obtained. The mice were euthanized in a CO_2_ chamber before the procedure. In a sterile environment, 10 mL of RPMI-1640 culture medium supplied with sodium bicarbonate was injected and collected from the peritoneum. Then, the recovered macrophages were seeded at a concentration of 1 × 10^5^ per well in a 96-well plate and kept in an incubator set at 37 °C and 5% CO_2_ for 24 h in RPMI-1640 medium supplemented with 2% FBS. After this period, cells were stimulated with the AMP (1.56–100 μM). After 24 h of interaction, the plate was centrifuged, and the supernatant recovered for further cytokine analysis. The remaining adherent cells were washed with PBS and 15 μL of lysis solution 10 × was added, from the Cytotox 96® (Promega, USA) kit, following the manufacturer’s instructions. The cellular viability was assessed through the dosage of lactate dehydrogenase (LDH) enzyme.

Humane peripheral blood was collected from healthy donors. The procedures to collect human peripheral blood cells were approved by the Ethics Committee on Human Research of Faculty of Health Sciences (CAAE n°. 36,760,920.3.0000.0030), University of Brasilia, from adult individual in accordance with the National Committee for Ethics in Research (CONEP) guidelines. Informed consent was obtained from all participants. All methods were carried out in accordance with relevant guidelines and regulations. The total volume was centrifuged at 800 g for 5 min and washed with PBS until the supernatant was clear. After that, two samples were prepared with PBS or sterile distilled water with 3% of erythrocytes added. In a 96-well plate, 50 μL of the AMP was added in a series dilution, ranging from 1.56 to 100 μM, and 50 μL of the erythrocyte samples. Thereafter, the plate was left for 1 h at room temperature. Following this period, the plate was centrifuged at 800 g for 5 min, the supernatant was collected for further analysis and the plate was read in a microplate reader at the wavelength of 540 nm^40^ .

### Immunomodulatory activity in vitro

Peritoneal macrophages were plated in a 96-well plate at the concentration of 1 × 105 cells/well and kept for 24 h in incubator at 37 °C with 5% CO2, for cell adhesion. The next day, the culture medium was exchanged, and the cells were stimulated with the peptide (6.25 μM and 25 μM) and/or LPS (500 ng/mL) with or without previous stimulation After the whole period of interaction, the supernatant was recovered and stored at -20 °C for cytokine dosage. In all assays of cytokine dosage, the samples were stored at − 20 °C. Cytokine levels were measured by ELISA (Enzyme-Linked Immunosorbent Assay) using Ready-SET-Go! ®(eBioscience™, Inc., Affymetrix, USA), following the manufacturer’s protocols.

### Data analysis

Analysis was carried out by using GraphPad Prism statistical analysis software version 8.0 (GraphPad*®*, La Jolla, California, USA). Thermal pain induction assay and pharmacological interaction assay results were submitted to two-way ANOVA followed by Tukey’s multiple comparisons test. The effect of the peptides on the FLIPR Ca2 + responses was evaluated through Pearson correlation. In vitro modulation model results were all submitted to one-way ANOVA followed by Tukey’s multiple comparisons test. The tolerance assay results were submitted to one-way ANOVA followed by Tukey’s multiple comparisons test for each day. Area under curve (AUC) was used to eliminate any possible pseudo-replication (while running one-way ANOVA) by the trapezoidal method, considering that the same animal was tested at different times. Values of *p* < 0.05 were considered statistically significant.

## Supplementary Information

Supplementary Information.

## Data Availability

The datasets generated during and/or analyzed during the current study are available from the corresponding author on reasonable request.
