# Supplementary material for: Exploring the therapeutic potential of an antinociceptive and anti-inflammatory peptide from wasp venom
Source: Sci Rep. 2023 Aug 1;13:12491. doi: 10.1038/s41598-023-38828-w (PMC10393941; doi:10.1038/s41598-023-38828-w)
Supplement: Supplementary file 1 — Supplementary Information. [file 41598_2023_38828_MOESM1_ESM.docx]

Exploring the Therapeutic Potential of An Antinociceptive and Anti-Inflammatory Peptide from Wasp Venom

Priscilla Galante^1^, Gabriel Avohay Alves Campos^1^, Jacqueline Coimbra Gonçalves^1^, Danubia B. Martins^2^, Márcia P. dos Santos Cabrera^2^, Marisa Rangel^3^, Luiza C. Coelho^5^, Karina S. Simon^5^, Veronica M. Amado^6^, Jessica de A. I. Muller^7^, Johannes Koehbach^8^, Rink-Jan Lohman^9^, Peter J. Cabot^9^, Irina Vetter^8^, David J. Craik^8^, Monica Cristina Toffoli-Kadri^7^, Victoria Monge-Fuentes^1^, Jair Trapé Goulart^1^, Elisabeth Ferroni Schwartz^1^, Luciano Paulino Silva^4^, Anamelia Lorenzetti Bocca^5^, Márcia Renata Mortari^1*^

^1^ Laboratory of Neuropharmacology, Department of Physiological Sciences, University of Brasília, Brasília, DF 70910-900, Brazil.

^2^ Department of Physics, IBILCE, São Paulo State University, São José do Rio Preto, SP 15054-000, Brazil.

^3^ Immunopathology Laboratory, Butantan Institute, Sao Paulo, SP 05503-900, Brazil

^4^ Laboratory of Nanobiotechnology, Embrapa Genetic Resources and Biotechnology, Brasília, DF 70770917, Brazil.

^5^ Laboratory of Applied Immunology, Department of Cell Biology, University of Brasilia, Brasilia, DF 70910-900, Brazil.

^6^ Faculty of Medicine and University Hospital of Brasília, University of Brasilia, Brasilia, DF 79910-900, Brazil.

^7^ Laboratory of Pharmacology and Inflammation FACFAN/Federal University of Mato Grosso do Sul, Campo Grande, Mato Grosso do Sul, 79070-900, Brazil.

^8^ Institute for Molecular Bioscience, Australian Research Council Centre of Excellence for Innovations in Peptide and Protein Science, The University of Queensland, Brisbane, Queensland, 4072, Australia.

^9^ School of Pharmacy, The University of Queensland, Brisbane, Queensland, 4072, Australia.

* Márcia Renata Mortari. **Email:**  mmortari@unb.br

**Supplementary Table S1**. Secondary structure elements (%) of Protonectin (PTN) and Protonectin-F (PTF) as determined by the deconvolution of CD and FTIR spectra.

| Secondary structure elements (%) | | | | | | | | | | | | |
| --- | --- | --- | --- | --- | --- | --- | --- | --- | --- | --- | --- | --- |
|  | H_2_O | | | | 40% TFE | | | | 8 mM SDS | | | |
|  | CD | | FTIR | | CD | | FTIR | | CD | | FTIR | |
|  | PTN | PTF | PTN | PTF | PTN | PTF | PTN | PTF | PTN | PTF | PTN | PTF |
| α-helix | 5 | 5 | - | - | 41 | 60 | 27 | 60 | 22 | 39 | 20 | 21 |
| β-sheet | 39 | 41 | - | 14 | 15 | 6 | - | - | 29 | 17 | - | - |
| turns | 21 | 21 | 49 | 38 | 19 | 14 | 27 | 13 | 18 | 16 | 24 | 44 |
| aggregated | ND | ND | 12 | 13 | ND | ND | 20 | 19 | ND | ND | 30 | 15 |
| random coil | 35 | 33 | 39 | 35 | 25 | 20 | 26 | 8 | 31 | 28 | 26 | 20 |

ND, not determined; -, absent;


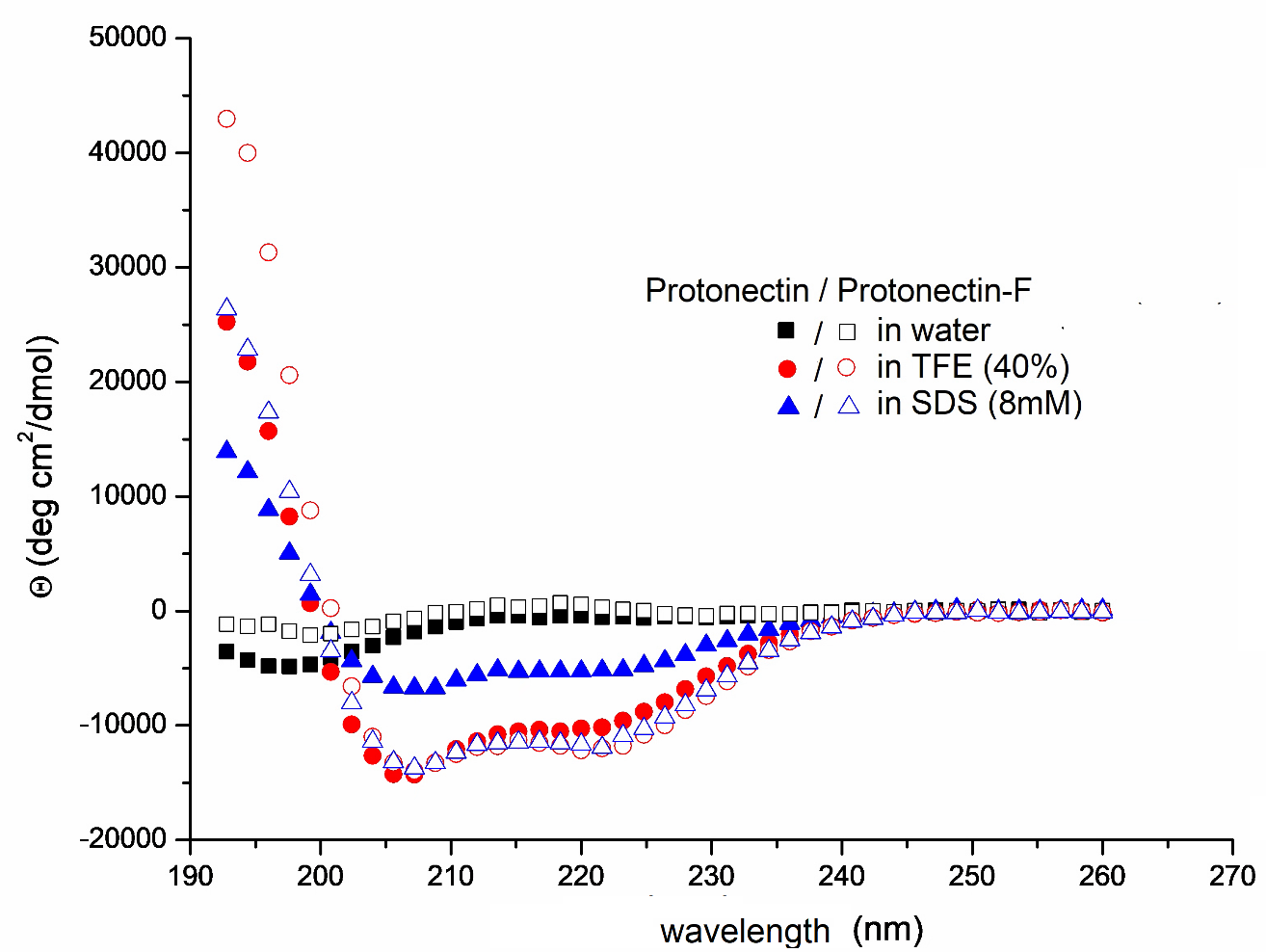


**Supplementary Figure S2.** CD spectra of protonectin and protonectin-F, obtained at 20 μM concentration, at 25 ˚C, in different environments. Spectra are the average of 10 accumulations obtained at 50 nm/min. No smoothing has been applied.

**Supplementary Figure S3**. Motor deficit evaluation on Rotarod model. Animals were administered 8 nmol or 16 nmol of protonectin, protonectin-F or morphine and challenged for three minutes on the Rotarod. When animals failed to stay on the equipment for the whole experiment, a failure was counted. Results were presented as a percentage of failure compared to the total animals of the group. Chi-square test, (*) significant effect in relation to vehicle group, p<0.05. # Significant effect in relation to protonectin-F, p<0.05.

**Supplementary Figure S4.** A- Hemolytic activity of 1-100 µM of protonectin-F on washed blood cells. Values were expressed as percentages compared to untreated control. B- Cytotoxicity of 1-100 µM of protonectin-F on peritoneal murine macrophages kept at 37 °C for 24 hours. The cellular viability was assessed through the dosage of lactate dehydrogenase (LDH) enzyme in the supernatant.

**Supplementary Figure S5.** Intracellular Ca^2+^ measurement in Fluo-4-loaded SH-SY5Y cells. **A**- Protonectin and Protonectin-F caused a dose-dependent increase in intracellular Ca^2+^ with EC50 of 57.87±1.33 µM and 21.51±0.43 µM, respectively. **B-C** Correlation between the peptide concentration and the maximum Ca^2+^ increase evoked by 50 µM Veratridine (Protonectin: Pearson r = 0.3275, p=0.3557; Protonectin-F: Pearson r = 0.2158, p=0.5493). **D-E** Correlation between the peptide concentration and the maximum Ca^2+^ increase evoked by 30µM Nicotine (Protonectin: Pearson r = -0.4821, p=0.2324; Protonectin-F: Pearson r = -0.6037, p=0.3645). Data are presented as mean ± SEM of n = 3 wells. Ca^2+^ fluorescence amplitudes were normalized to the baseline.


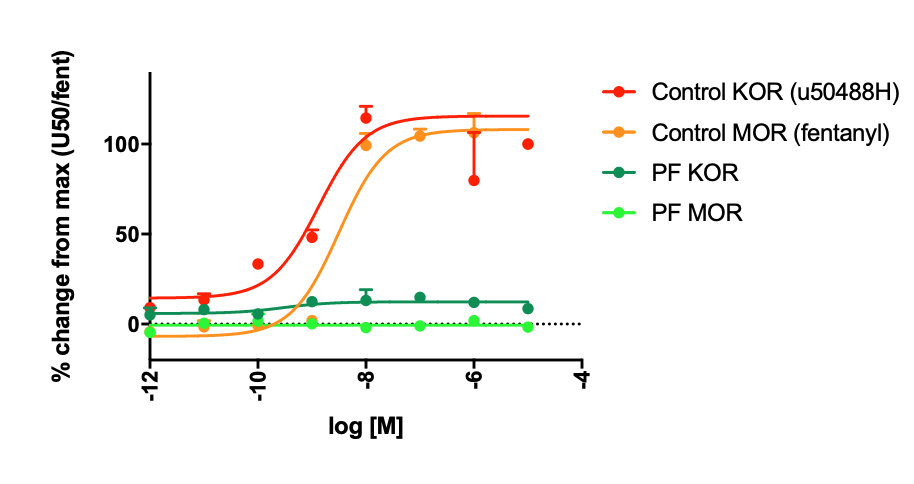


**Supplementary Figure S6.** Potential activity of the peptide protonectin-F (PF) at opioid receptors μ (MOR) and κ (KOR).


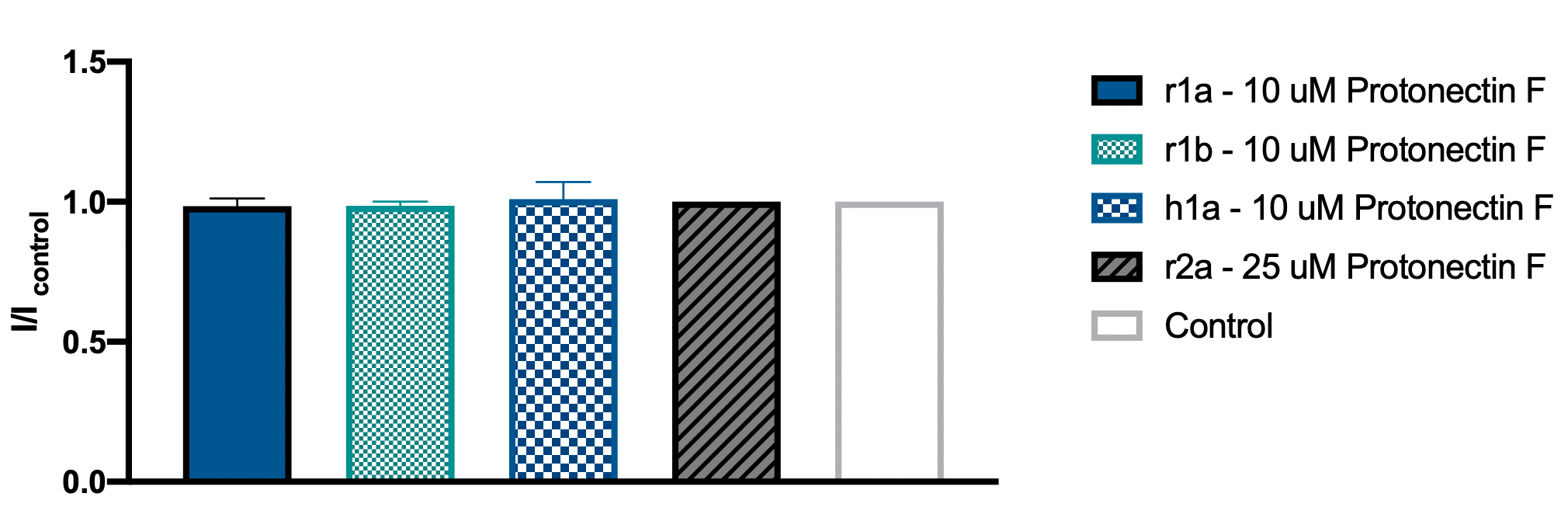


**Supplementary Figure S7.** Activity of protonectin-F (PF) on acid-sensing ion channels (rat ASIC1a (r1a), rat ASIC1b (r1b), human ASIC1a (h1a), and rat ASIC2a (r2a)) as assessed using two-electrode voltage clamp electrophysiology on *Xenopus laevis* oocytes heterologously expressing ASICs. N=3-4, data expressed as mean ± SEM.
